# Mitochondrial dynamics-related genes DRP1 and OPA1 contributes to early diagnosis of cognitive impairment in diabetes

**DOI:** 10.1186/s12877-023-04156-x

**Published:** 2023-08-10

**Authors:** Mengqian Liu, Chen Gong, Xiaozhu Shen, Yi Jiang, Yiwen Xu, Wen Zhong, Yujiao Chen, Nan Dong, Jingxian Liao, Ning Yin

**Affiliations:** 1 Department of Geriatrics, Lianyungang Hospital Affifiliated to Jiangsu University (Lianyungang Second People’s Hospital), Lianyungang, China; 2 Department of Geriatrics, Bengbu Medical College Clinical College of Lianyungang Second People’s Hospital, Lianyungang, China; 3 Department of Laboratory Medicine, Lianyungang Second People′s Hospital, Lianyungang, China; 4 Department of Neurology, Suzhou Industrial Park Xinghai Hospital, Suzhou, China

**Keywords:** Mitochondrial dynamics-related genes, Drp1, Opa1, Diabetes, Cognitive impairment

## Abstract

**Background and aim:**

DRP1 and OPA1 play important roles in mitochondrial fusion and fission. However, the role of DRP1 and OPA1 amplification in mitochondrial cognitive impairment has not been reported. This study aimed to investigate the relationship between DRP1 and OPA1 and the risk of cognitive impairment.

**Methods:**

In this study, 45 elderly patients with diabetes admitted to the Lianyungang Second People’s Hospital from September 2020 to January 2021 were included. The patients were divided into normal group, mild cognitive impairment group and dementia group by using MMSE score, and the clinical characteristics of the three groups were compared. The amplification multiples of the two genes’ DNA were calculated by ΔΔCT and defined as 2^− K^. Spearman rank correlation was used to analyze the correlation between the DNA amplification multiples of patients’ DRP1 and OPA1 and AD8 and MoCA scores. The sensitivity and specificity of DNA amplification multiples of DRP1 and OPA1 to predict clinical outcomes of diabetic cognitive impairment were evaluated using Receiver operator characteristic (ROC) curves. Multiple logistic regression was used to evaluate the relationship between DNA amplification factor of DRP1 and OPA1 and cognitive function.

**Results:**

DRP1(2^− K^) and OPA1(2^− K^) significantly increased and decreased in dementia and MCI groups compared with the normal group (P ≤ 0.001). The DNA amplification factor of DRP1 was positively correlated with AD8 score and negatively correlated with MoCA score (P < 0.001). The DNA amplification factor of OPA1 was positively correlated with the MoCA score (P = 0.0002). Analysis of ROCs showed that the DNA amplification factor of OPA1 had a higher predictive value for dementia (P < 0.0001), and that it had a higher predictive value when used in combination with DRP1. Multiple logistic regression results showed that increased DNA amplification in DRP1 was associated with increased risk of dementia (OR 1.149;95%CI,1.035–1.275), and increased DNA amplification in OPA1 was associated with decreased risk of MCI (OR 0.004;95%CI,0.000-0.251) and dementia (OR 0.000;95%CI,0.000-0.134).

**Conclusion:**

DNA amplification multiples of DRP1 and OPA1 are associated with the risk of dementia in elderly patients and may serve as potential biomarkers.

## Introduction

Diabetes is a common and frequently occurring disease in the elderly. China, which is the most populous country, ranks number one with an estimate of 109.6 million adults with diabetes. The prevalence of diabetes in China has sharply increased over the past three decades [1]. Cognitive impairment and dementia (including Alzheimer’s disease,AD) are increasingly recognized as common complications of type 1 and type 2 diabetes mellitus (T1 and T2DM) [2]. Dementia is the most progressive stage of cognitive dysfunction, with impairment of multiple cognitive domains that interfere with daily life activities [3]. Impaired insulin signaling, increased expression of advanced glycation end products (AGEs), chronic inflammation, oxidative stress, and mitochondria dysfunction contribute to the development of DM-associated neurodegeneration and cognitive decline [4].

DRP1, also known as dynamic-related protein 1, is involved in mitochondrial fission. The overexpression of DRP1 protein in embryonic hippocampal neurons can change the mitochondrial structure and impair the formation of dendritic branches [5]. The inhibition of DRP1 protein can restore mitochondrial density, increase ATP algebra, prevent mitochondrial membrane potential loss, and protect neurons from ischemic stroke, further confirming the importance of DRP1 in mitochondrial structure and cell function [6]. OPA1, also known as optic nerve atrophy protein 1, is a dynamin-related GTP enzyme located in the mitochondrial intima. It was essential for mitochondrial intima fusion and was originally identified by screening for genetic mutations in autosomal dominant optic nerve atrophy [7]. However, current studies have not clarified the relationship between DRP1 and OPA1 expression and cognitive dysfunction in diabetes mellitus.

In this study, we assessed DNA amplification multiples of DRP1 and OPA1 in patients with normal cognitive function, mild cognitive impairment (MCI), and dementia;investigated their potential value as biomarkers for MCI and dementia; and explored the relationship between DNA amplification multiples of DRP1 and OPA1 and the risk of cognitive impairment.

## Methods

### Patients and samples

The participants were recruited after receiving their written informed consent and approval from the ethics committee of Lianyungang Second People’s Hospital. Elderly patients aged more than 60 years treated in the Lianyungang Second People’s Hospital from September 2020 to January 2021 were recruited for this study. All the programmes and procedures we studied were carried out in accordance with the Declaration of Helsinki. The study was ethically approved by the Lianyungang Second Hospital (No. 2016-036-01).

The inclusion criteria were as follows: (1) meeting the diagnostic criteria of aged patients with type 2 diabetes, (2) age ≥ 60 years, and (3) informed consent and voluntary cooperation of patients. The exclusion criteria were as follows: (1) patients having a history of brain injury, cerebral infarction, severe hypoglycemia, and severe diabetic ketosis or ketoacidosis, (2) patients with serious y diseases of the cardiovascular system, liver, kidney, and hematopoietic system, (3) patients with related neuropsychiatric history, depression, and intake of antidepressants, (4) patients having other diseases or medical history causing central nerve injury, and (5) patients with secondary diabetes.

The severity of simple dementia was graded according to the MMSE score: illiteracy ≤ 17 points, primary school ≤ 20 points, middle school (including technical secondary school) ≤ 22 points, and university (including junior college) ≤ 23 points. Dementia was classified as follows: mild, ≥ 21 points; moderate, 10–20 points; and severe, ≤ 9 points. Based on their cognitive functions, the patients were divided into 3 groups: 15 in the dementia group, 15 in the MCI group, and 15 in the normal cognitive function group. The MMSE, AD8 and MoCA scores were evaluated by 2 experienced neurologists who had been systematically trained (unaware of other clinical data). The reliability and validity of MoCA scale were 0.97 and 0.88 respectively [8]; the reliability and validity of MoCA scale were 0.97 and 0.88 respectively [9]. The reliability and validity of MoCA scale were 0.97 and 0.88 respectively.

The reliability and validity of the AD8 scale were 0.96 and 0.78.The AD8 score assesses cognitive functions such as memory and orientation by asking the patient, which has a total score of 8, with 0–1 being normal cognitive function and ≥ 2 being cognitive impairment. The MoCA score provides a rapid screen for mild cognitive functioning domains, with a total score of 30 and ≥ 26 being normal. The outcome variable of this study was cognitive function. Then, 5 mL of fasting EDTA-anticoagulated whole blood of all participants was collected in the morning, placed in a 1.5-mL centrifuge tube (without RNA enzyme and autoclave), and stored at − 80℃ in the refrigerator.

### Basic data collection

During enrolment, the medical histories were taken and routine physical examinations of the participants were performed by experienced physicians. The medical history included age and sex. The laboratory tests included creatinine (Cr), blood urea nitrogen (BUN), alanine transaminase (ALT), aspartate aminotransferase (AST), cholesterol (TC), triglyceride (TG), low density lipoprotein-cholesterol (LDL), high density lipoprotein-cholesterol (HDL), albumin (ALB), hemoglobin A1C (HbA1c), triiodothyronine (T3), thyroxine (T4), thyroid-stimulating hormone (TSH).

#### Total RNA extraction by the Trizol method

The whole blood (200 µL) was taken and mixed with 800 µL of the RNA extract. Further, 250 µL of trichloromethane was added and the centrifuge tube was reversed for 15s, mixed thoroughly, and allowed to stand for 3 min. The mixture was centrifuged at 12,000 rpm at 4℃ for 10 min. The supernatant was transferred to a new centrifugal tube, and 0.8 times the volume of isopropyl alcohol was added and mixed inversely at − 20℃ for 15 min. After centrifugation at 12,000 rpm at 4℃ for 10 min, the white precipitate at the bottom of the tube was RNA. The liquid was removed, and 1.5 mL of 75% ethanol was added to wash the precipitate. The suspension was centrifuged at 12,000 rpm at 4℃ for 5 min. The liquid was sucked clean, and the centrifuge tube was placed on the ultra-clean table and blown for 3 min. Next, 15 µL of RNA-free water was added to dissolve the RNA and incubated at 55℃ for 5 min. A NanoDrop2000 spectrophotometer was used to detect RNA concentration and purity. Further, 2.5 µL of RNA solution to be measured on the detection base after the instrument blank zero, put down the sample arm, use the software on the computer to start absorbance value detection. The RNA with excessive concentration was diluted at an appropriate proportion to reach the final concentration of 100–500 ng/µL.

#### Reverse transcription

A polymerase chain reaction (PCR) tube was taken, and 10 µL of RNA solution was added, followed by 0.5 µL of Oligo (dT)18 primer and 0.5 µL of random hexamer primer. The mixture was supplemented with non-ribonuclease deionized water to a volume of 15 µL. It was kept warm at 65℃ for 5 min on the PCR instrument and quickly placed on ice for cooling. Then, 4 µL of 5× reaction buffer and 1 µL of Servicebio RT Enzyme Mix were added, suctioned, and mixed. The PCR apparatus was kept at 42℃ for 60 min, and the reverse transcriptase was inactivated at 70℃ for 5 min.

#### Quantitative real-time PCR

The primer sequences (5’-3’) used for qPCR were as follows: DRP1: upstream 5’-TGGGGCGCCGACATCA-3'; downstream 5’-GCTCTGCGTTCCCACTACGA-3'. OPA1: upstream 5’-GTGCTGGCCCGCCTAGAAA-3'; downstream 5’-TGACAGGCACCCGTACTCAGT-3'. The total reaction volume was 20 µL, including 2 µL of primer, 2 µL of reverse transcripts, 6 µL of ddH_2_O, and 2× PCR reaction buffer. PCR cycle conditions were as follows: pre-denaturation at 95℃ for 10 min; denaturation at 95℃ for 15 s, annealing at 60℃ for 60 s, extension at 72℃ for 90 s, and a total of 40 cycles. Finally, it was extended at 72℃ for 5 min.

#### Computing method

Calculated by the ΔΔCT method: A = CT (target gene, sample to be tested) – CT (internal standard gene, sample to be tested); B = CT (target gene, control sample) – CT (internal standard gene, control sample); k = A – B; expression multiple = 2^− k^.

### Statistical analysis

**Fig. 1:**
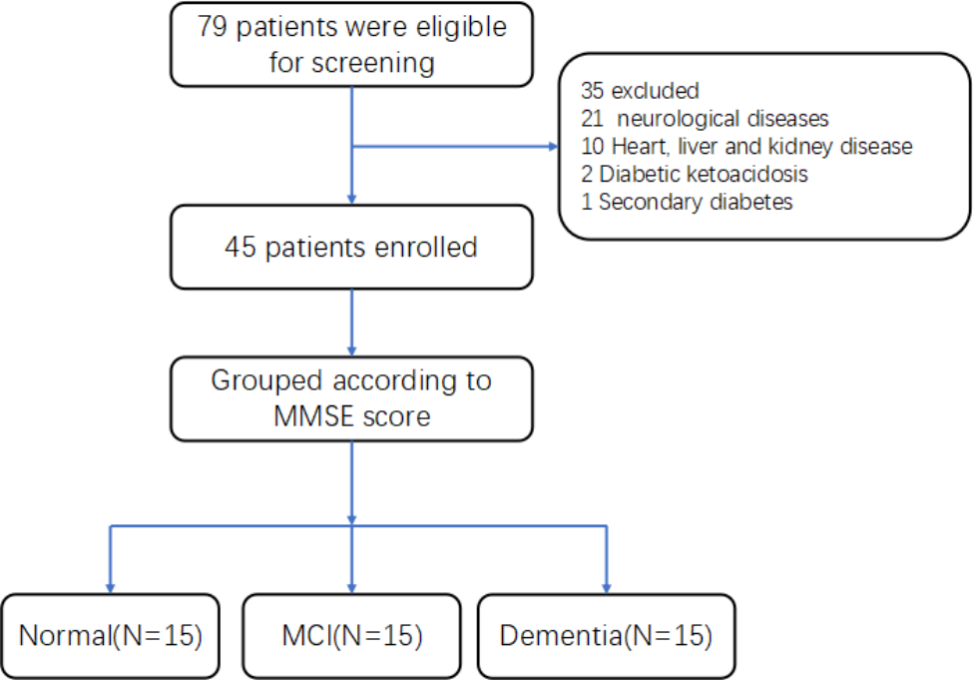
Study protocol flowchart

All data were analyzed using the SPSS software (IBM SPSS Statistics for Windows, version 22.0; IBM Corp, NY, USA) and GraphPad Software (GraphPad Prism for Windows, version 9.0.0; CA, USA). The minimum sample size was estimated by PASS. Univariate analysis of variance was used to calculate the sample size, with Power = 0.9, Alpha = 0.05, Sm = 14.48, S = 24.78, and The minimum sample size was 42, with 14 cases in each group. The baseline characteristics were analyzed using the following methods. The Kolmogorov–Smirnov test was used to assess the normality of numerical variables. Median and interquartile range (IQR) were used to describe continuous variables with non-normal distribution. The normal-distribution data were analyzed by analysis of variance. The data were expressed as mean ± standard deviation (SD), including ALT, TC, median LDL, median HbA1c, median T3, and median T4. The non-normal-distribution data were analyzed by the Kruskal–Wallis test, including AD8 score, MoCA score, age, Cr, TG, BUN, AST, median HDL, median ALB, median TSH, DRP1 (2^− K^), and OPA1 (2^− K^). Fisher’s exact test or the chi-square test was used to compare categorical variables as appropriate, including sex (Table 1; Fig. 1). ROCs were used to find out the better clinical indicators and assess the sensitivity and specificity of DRP1 and OPA1 gene amplification multiples for predicting the clinical outcome of normal, MCI, and dementia groups. The Youden index was used to calculate the optimal diagnostic cutoff point, optimal sensitivity, and specificity of each variable. The Youden index was equal to the value of sensitivity minus (1 – specificity). Moreover, Spearman rank correlation was used for the correlation analysis of DRP1/OPA1 gene amplification multiples and AD8/MoCA scores in patients (Fig. 3). Multiple logistic regression analyses were used to determine how plasma DNA amplification multiples of DRP1 and OPA1 affected cognitive function in diabetes and adjusted for confounding factors (Fig. 4). A P value < 0.05 (bilateral) indicated a statistically significant difference.

**Table 1:** Baseline characteristics of the studied patient population (N = 45)

Characteristics	Normal (*n* = 15)	MCI (*n* = 15)	Dementia (*n* = 15)	*F*/X *χ*^2^	*P* value
Median age (IQR), year	74(65.5–78)	79(69.5–87)	83(75–87)	4.667	0.097
Gender, no. (%)					0.181
Male	9(60)	7(46.7)	4(26.7)		
Female	8(53.3)	6(40)	11(73.3)		
Median Cr (IQR), mmol/L	68 (55.5–77)	65 (55–87.5)	66 (58–108)	1.006	0.605
Median BUN (IQR), mmol/L	6.00 (5.30–7.00)	7.31 (5.90–10.40)	7.30 (5.90–11.15)	3.140	0.208
ALT (SD), U/L	28.47 ± 16.89	28.80 ± 14.84	25.87 ± 13.57	0.618	0.846
AST (IQR), U/L	21 (18.5–23.5)	26 (21–38)	22(18–27)	5.285	0.071
Median TC (SD), mmol/L	4.50 ± 0.89	4.71 ± 0.93	3.99 ± 1.03	2.291	0.114
Median TG (IQR), mmol/L	1.29 (0.96–2.13)	1.56 (1.33–1.94)	1.54 (1.32–2.00)	0.955	0.62
Median HDL (IQR), mmol/L	1.25 (1.20–1.50)	1.16 (1.08–1.31)	1.12 (1.02–1.34)	3.957	0.138
Median LDL (SD), mmol/L	3.09 ± 0.82	3.22 ± 0.79	2.40 ± 0.86	4.257	0.021
Median ALB (IOR), mmol/L	40.9 (34.95–44.5)	35.7 (32,65–42.2)	37.2 (33.2–41.4)	2.811	0.245
Median HbA1c(SD), %	7.64 ± 0.95	7.30 ± 1.03	8.48 ± 1.40	6.135	0.022
Median T3 (SD), mmol/L	5.13 ± 1.04	5.06 ± 0.80	4.50 ± 0.60	2.552	0.090
Median T4 (SD), mmol/L	12.26 ± 1.92	11.00 ± 1.47	12.08 ± 2.11	2.041	0.143
Median TSH (IQR), mmol/L	1.46 (1.10–1.98)	1.81 (1.11–2.58)	1.59 (1.30–1.87)	0.715	0.699
DRP1 (2^− K^) (IQR)	12.91 (8.3–27.91)	30.71 (20.66–35.97)	39.40 (35.16–86.43)	14.517	0.001
OPA1 (2^− K^) (IQR)	0.90 (0.50–1.10)	0.21 (0.16–0.45)	0.15 (0.09–0.23)	20.802	0.000

## Results

### Baseline characteristics of participants

Among the 79 eligible patients, a total of 45 were eventually enrolled in the study. The clinical characteristics of all participants were summarized in Table 1. The HbA1c and DRP1 (2^− K^) levels were significantly higher, while LDL and OPA1 (2^− K^) levels were significantly lower in the dementia group compared with the normal and MCI groups. No significant differences were found in gender; age; Cr, BUN, TC levels; and other clinical features among the three groups.

### Comparison of the DNA amplification multiples of three groups of DRP1 and OPA1

**Fig. 2:**
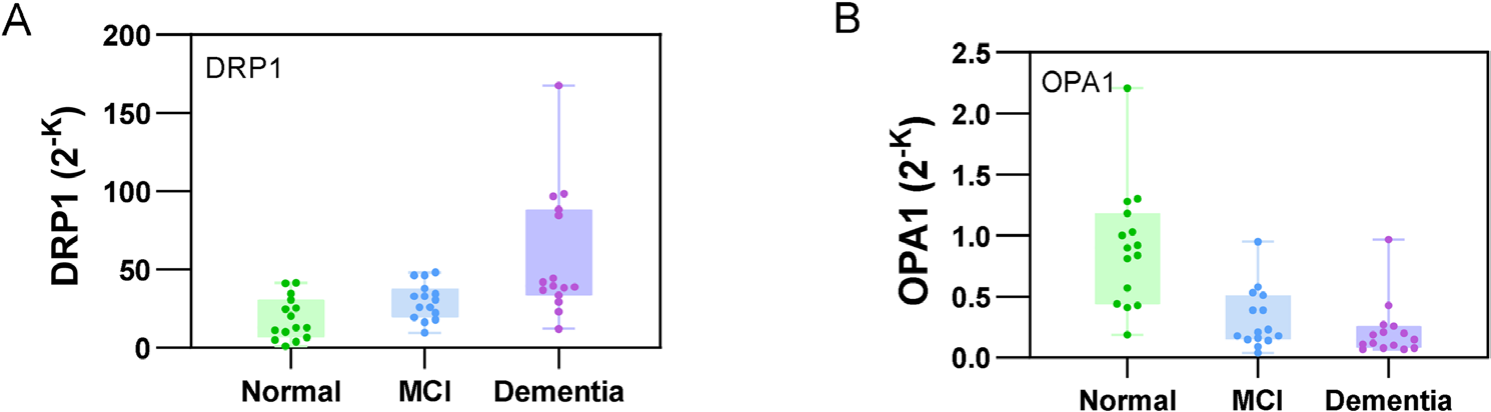
Gene amplification multiples of the normal, MCI, and dementia groups. (**A**) Higher DRP1 gene amplification multiples in patients with dementia, P < 0.001, Kruskal–Wallis test. (**B**) Lower OPA1 gene amplification multiples in patients with dementia, P < 0.001, Kruskal–Wallis test

The DNA amplification multiples of DRP1 amplification significantly increased and the DNA amplification multiples of OPA1 amplification significantly decreased in the MCI and dementia groups compared with the normal group (DRP1: normal vs. MCI vs. dementia: 12.91 [8.3–27.91] vs. 30.71 [20.66–35.97] vs. 39.40 [33.56–88.16]; OPA1: normal vs. MCI vs. dementia: 0.90 [0.50–1.10] vs. 0.21 [0.16–0.45] vs. 0.15 [0.09–0.23]) (Fig. 2). The baseline characteristics of the three groups were shown in Table 1.

### Association of DRP1/OPA1 gene amplification multiples with MCI/dementia

**Fig. 3:**
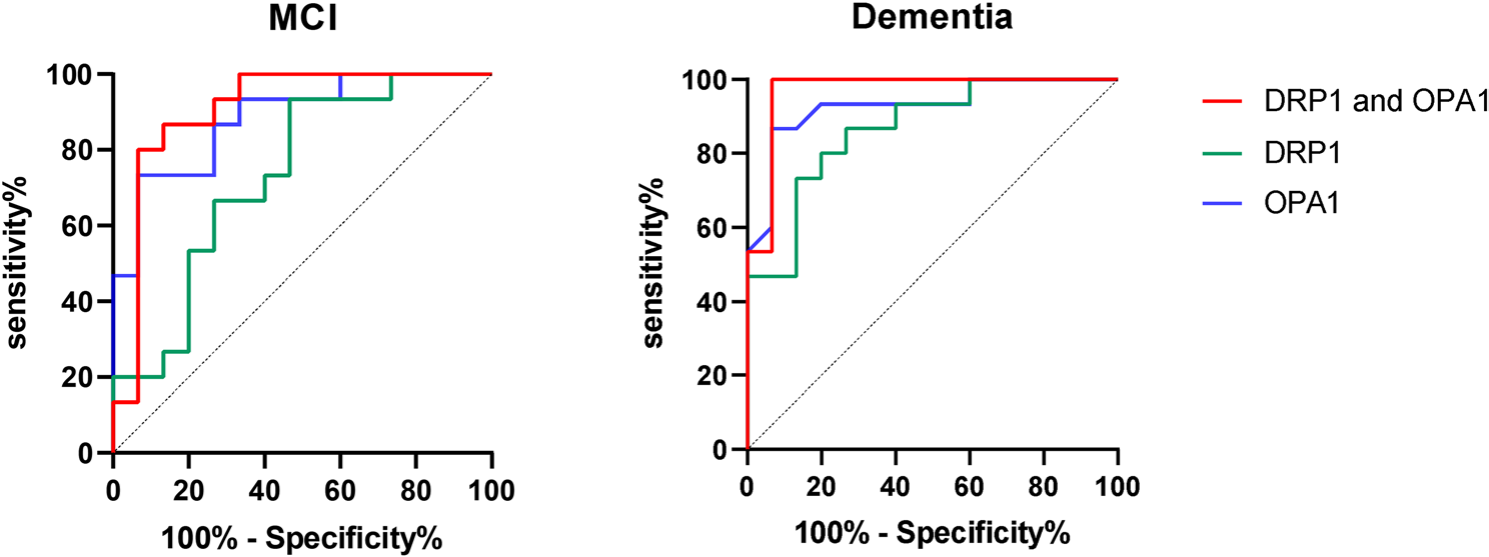
ROC for evaluating the association between DRP1/OPA1/DRP1 and OPA1 gene amplification multiples and MCI/dementia. **MCI** DRP1: AUC = 0.7333; 95% CI: 0.5507–0.9160; P = 0.0294 OPA1: AUC = 0.8844; 95% CI: 0.7665–1.0000; P = 0.0003 DRP1 and OPA1: AUC = 0.9067; 95% CI: 0.7866–1.0000; P = 0.0001 **Dementia** DRP1: AUC = 0.8667; 95% CI: 0.7391–0.9943; P = 0.0006 OPA1: AUC = 0.9289; 95% CI: 0.8336–1.0000; P < 0.0001 DRP1 and OPA1: AUC = 0.9689; 95% CI: 0.9055–1.0000; P < 0.0001

ROC analysis showed that the combined predictive value of DRP1 and OPA1 was higher than that of the individual predictive values in both MCI and dementia groups (Fig. 3). The AUC, optimal cutoff value, sensitivity, and specificity of DRP1 and OPA1 amplification in the differential diagnosis of normal cognitive function, MCI, and dementia are shown in Tables 2 and 3. The amplification of OPA1 had a better accuracy for the diagnosis of dementia (AUC = 0.9289, P < 0.0001). When the optimal cutoff value of OPA1 amplification was 0.34, the sensitivity was 86.7, and the specificity was 93.3. DRP1 amplification also had a good diagnostic value for dementia (AUC = 0.8667, P = 0.0006). The amplifications of OPA1 and DRP1 also had a certain diagnostic value for MCI (AUC = 0.7333 and 0.8844; P = 0.0294 and 0.0003).

**Table 2:** ROC analysis for the differential diagnosis between normal cognitive function and MCI

Variable	AUC	*P* Value	95% CI		Boundary value	Sensitivity (%)	Specificity (%)
			Lower bound	Upper bound			
DRP1 (2^− K^)	0.7333	0.0294	0.5507	0.9160	14.64	93.3	53.3
OPA1 (2^− K^)	0.8844	0.0003	0.7665	1.0000	0.4	73.3	93.3

**Table 3:** ROC analysis for the differential diagnosis between normal cognitive function and dementia

Variable	AUC	P Value	95% CI		Boundary value	Sensitivity (%)	Specificity (%)
			Lower bound	Upper bound			
DRP1 (2^− K^)	0.8667	0.0006	0.7391	0.9934	32.09	80	80
OPA1 (2^− K^)	0.9289	0.0000	0.8336	1.0000	0.34	86.7	93.3

### Correlation between DRP1/OPA1 gene amplification multiples and AD8/MoCA scores

**Fig. 4:**
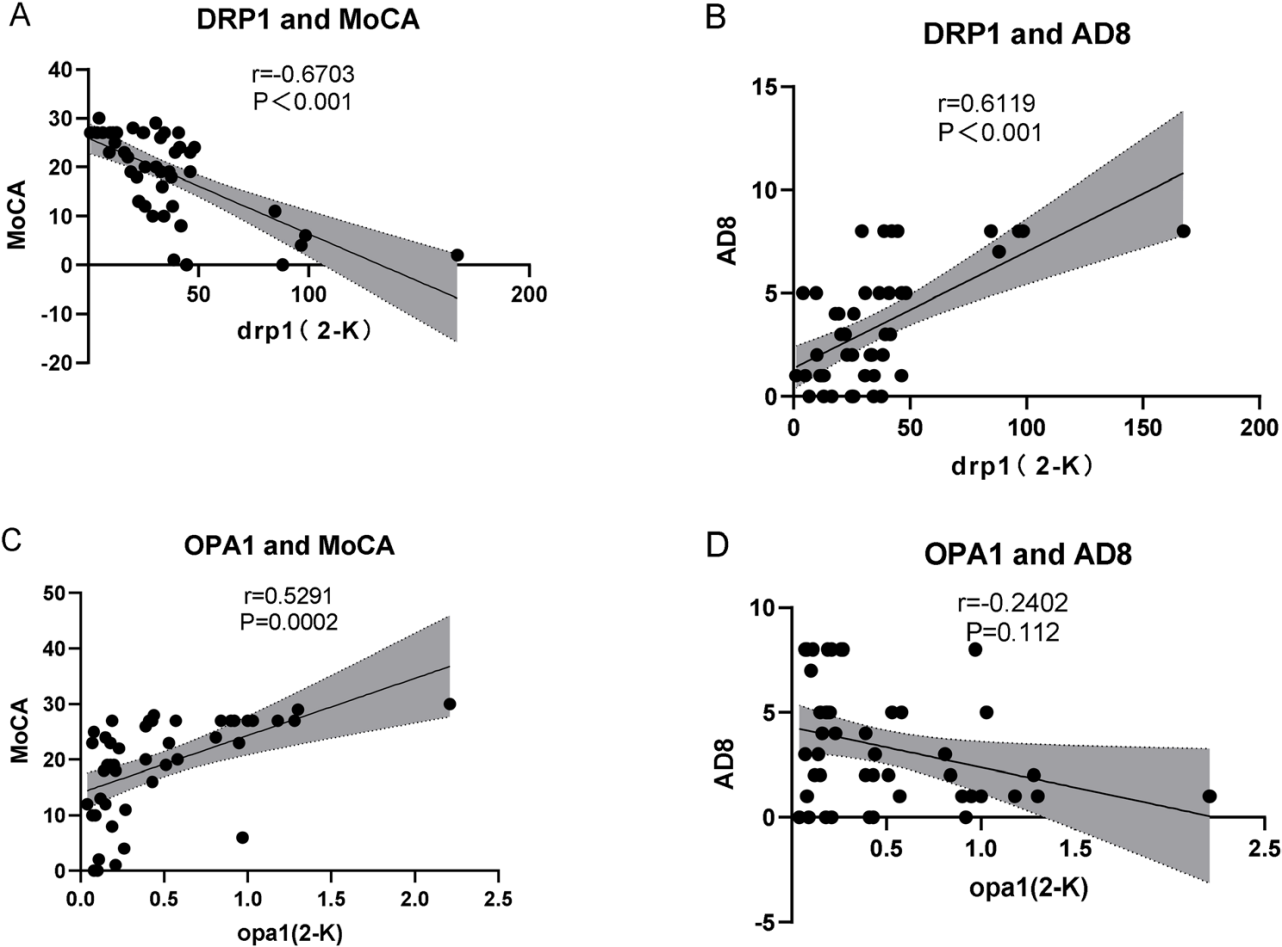
Correlation between DRP1/OPA1 gene amplification multiples and AD8/MoCA scores in patients evaluated using Spearman’s correlation coefficient

We further evaluated the correlation between DRP1/OPA1 gene amplification and AD8/MoCA score as a risk assessment criterion for dementia. DRP1 gene amplification was positively correlated with the AD8 score and negatively correlated with the MoCA score, and the difference was statistically significant. OPA1 gene amplification was positively correlated with the MoCA score, but there was no significant correlation with AD8 scores (Fig. 4).

### Multiple logistic regression analysis with MMSE score as dependent variable

The cognitive impairment outcome (MMSE score) of patients was selected as the dependent variable to study the relationship between the DNA amplification factor of DRP1 and OPA1, the results of which were shown in Table 4. Multiple logistic regression model 0 showed that DNA amplification factor of DRP1 was independently correlated with DM-related dementia(P = 0.009), and amplification factor of OPA1 was independently correlated with both diabetes-related MCI (P = 0.007) and dementia(P = 0.002), and this effect persisted after adjusting for HbA1c and LDL in Model 1. As shown in Model 1, the increased DNA amplification factor of DRP1 was associated with an increased risk of dementia (OR 1.149;95%CI,1.035–1.275; P = 0.009), and increased DNA amplification in OPA1 was associated with decreased risk of MCI (OR 0.004;95%CI,0.000-0.251; P = 0.009) and dementia (OR 0.000;95%CI,0.000-0.134; P = 0.010).

**Table 4:** Multiple logistic regression analysis with MMSE score as dependent variable

		Variables	B	SE	Wald χ^2^	P	OR	95%CI
Model0	MCI	DRP1(2^− K^)	0.064	0.043	2.204	0.138	1.066	0.980–1.160
		OPA1(2^− K^)	-5.229	1.939	7.276	0.007	0.005	0.000-0.239
	Dementia	DRP1(2^− K^)	0.134	0.051	6.870	0.009	1.144	1.034–1.265
		OPA1(2^− K^)	-9.230	3.044	9.197	0.002	0.000	0.000-0.038
Model1	MCI	DRP1(2^− K^)	0.065	0.043	2.245	0.134	1.067	0.980–1.162
		OPA1(2^− K^)	-5.467	2.084	6.882	0.009	0.004	0.000-0.251
	Dementia	DRP1(2^− K^)	0.139	0.053	6.779	0.009	1.149	1.035–1.275
		OPA1(2^− K^)	-8.478	3.300	6.599	0.010	0.000	0.000-0.134

Model 0: Unadjusted;

Model 1: Adjusted for HbA1c and LDL

## Discussion

The results of this study showed that plasma DRP1 amplification increased and OPA1 amplification decreased in patients with diabetes than in those with MCI and dementia. The multiples of DRP1 and OPA1 amplification were positively correlated with AD8 and MoCA scores, which were evaluation tools for screening cognitive impairment and dementia. Therefore, the amplification index of DRP1 and OPA1 in peripheral blood were associated with the occurrence of diabetic neurocognitive impairment and dementia, and had a certain predictive effect on their occurrence.

Previous studies proved that mitochondria played an important role in the survival and function of neurons, and their dysfunction was related to degenerative neuropathy, such as AD [10]. The pathogenesis involved in the neural degeneration process were basically as follows. (1) In patients with neurodegenerative diseases, complexes were deficient in mitochondria in the substantia nigra and platelets, resulting in the loss of electron transport chains that affected neuronal function, mainly in AD complexes I [11–13]. (2) Fusion and fission were the important processes maintaining mitochondrial dynamics [14]. Excessive mitochondrial fission affected energy production by affecting the assembly of oxidative phosphorylation complexes, while reduced mitochondrial fusion inhibited mitochondrial repair by increasing the proportion of dysfunctional organelles. Mitochondrial fusion/fission imbalance might eventually lead to synaptic dysfunction [15].

Thus, poor blood glucose control promotes the accumulation of AGEs, and their accumulation may lead to molecular and cellular damage that contributes to diabetes-induced brain aging. In addition, the role of oxidative stress in the pathogenesis of cognitive dysfunction in rats should not be ignored [16]. Oxidative stress and reduced antioxidant defenses create a deleterious combination that disrupts cell function and damages cells, leading to the loss of synapses and cell death. At the same time, hyperglycemia can also activate a variety of signaling pathways, leading to increased ROS production and insulin resistance [17].

DRP1 and OPA1 are two factors that play important roles in mitochondrial fusion and cleavage. DRP1 is mainly involved in mitochondrial fission. Increased protein expression and/or phosphorylation of the Serine 616 residue of DRP1 has been reported in neurons that undergo neuroexcitatory toxicity induced by oxygen/glucose deprivation [10, 18, 19]. Also, recent studies revealed that Aβ interacted with DRP1, with a subsequent increase in free radical production. This, in turn, activated DRP1, resulting in excessive mitochondrial fragmentation and defective transport of mitochondria to synapses. It also provided reduced synaptic ATP and ultimately led to synaptic dysfunction [20]. Further studies proved that DRP1 might lead to excessive mitochondrial fragmentation, mitochondrial and synaptic defects, and ultimately neuronal damage and cognitive decline through interaction with phosphorylated Tau [21]. Consistent with these findings, Hu et al. treated mice with diabetes with the DRP1 inhibitor Mdivi-1 to prevent mitochondrial fission, inhibit neuronal death, and restore cognitive function, suggesting that DRP1 protein played a protective role in neuronal function [6]. It was speculated that DRP1 was a marker of cognitive dysfunction and used as a marker in Huntington’s disease [22], FOXP1 syndrome [23], and other complicated forms of dementia.

OPA1 protein is a kinin-related GTPase involved in mitochondrial fusion, morphology, and apoptosis [24]. Its mutation and deletion are related to a variety of neurodegenerative diseases, such as AD and Parkinson’s syndrome [25]. Bertholet et al. found that the knockdown of OPA1 in mice resulted in mitochondrial fragmentation, reduced mitochondrial number in dendrites and axons, and defective mitochondrial bioenergetics, which might be caused by a decrease in presynaptic and postsynaptic protein expression and synaptic number [26]. Kushnareva et al. showed that reduced OPA1 expression resulted in mitochondrial fragmentation, loss of oxidative phosphorylation, decreased ATP production, decreased mitochondrial Ca^2+^ retention, and sensitivity to apoptotic damage in retinal neurons [27]. This might be explained by the fact that the loss of OPA1 reduced cytosolic Ca^2+^ buffering and sensitized retinal ganglion cells to excitotoxic damage induced by glutamate exposure, suggesting a possible role of OPA1 in synaptic transmission [27]. A previous study demonstrated that the heterozygous loss of OPA1 resulted in premature age-related loss of spines in hippocampal pyramidal CA1 (the region in the hippocampal circuit) neurons and a reduction in synaptic density in the hippocampus. The loss was associated with subtle memory deficits in both spatial novelty and object recognition [25]. In general, OPA1 protein reduction affected neuronal function mainly by affecting presynaptic proteins, postsynaptic proteins, and synaptic transmission, and then caused neuropathy. In a study investigating diabetic neuropathy, OPA1 was found to be significantly downregulated in motor neurons grown under HG conditions and in lumbar spinal cord tissues of rats with type 1 diabetes [28]. These findings indicated that HG- or diabetes-induced OPA1 downregulation could result in significant negative consequences related to diabetic complications. In addition, Kim et al. found that diabetes induced a decrease in OPA1 expression, which increased cytochrome C release and promoted mitochondrial fragmentation in retinal vascular cells in rodents with diabetes [29]. These findings were consistent with the results of decreased OPA1 expression in patients with dementia in our study.

In addition, mitochondrial dynamics is associated with the production of reactive oxygen species in the presence of high glucose. Previous studies have shown [30], that mitochondria rapidly fragment in a high-glycemic environment, accompanied by the production of reactive oxygen species. However, the mechanism of how mitochondria affect reactive oxygen species has not been clarified. Therefore, we hypothesized that hyperglycemia affected mitochondrial dynamics by affecting the amplification of DRP1 and OPA1, thus affecting the progression of diabetic cognitive impairment, and further experiments are still needed for verification.

In the comparison of clinical data among the three groups, we found that the difference of LDL in the three groups was also statistically significant. A study in 2015 [31] found that patients with hypercholesterolemia had higher levels of LDL oxidation than those in the control group, which can disrupt the microvascular barrier in the brain, leading to cognitive decline. In addition, insulin resistance in patients with T2DM can cause oxidative stress [32], so LDL may further affect cognitive function through peroxidation stress pathway. However, there are few studies on this aspect at present, so there is no conclusion. In addition, HbA1c was also different among the three groups, and its serum level in dementia patients was significantly higher than that in the normal group and the MCI group. A study in 2019 [33] found that elderly diabetic patients with HbA1c ≥ 7.0% had a 38% increased risk of cognitive impairment. Long-term hyperglycemia may lead to the thickening of the vascular basement membrane, reduced circulation of blood in the brain, direct damage to neurons, and thus cognitive dysfunction [34]. Although HbA1c and LDL play a role in diabetes-related cognitive impairment, the effect of DNA amplification of DRP1 and OPA1 on diabetic cognitive function remained in our study after their effects were excluded.

However, our study found a more statistically significant correlation between DRP1 and AD8 and MoCA scores, possibly because excessive mitochondrial fission was a major component of the pathology that caused AD and other degenerative diseases [35].

Our study found that the combination of DRP1 and OPA1 amplification index was significantly more predictive of MCI and dementia than either indicator alone [MCI (AUC): DRP1 + OPA1 vs. DRP1 and OPA1 = 0.9067 vs. 0.7333 and 0.8844; dementia (AUC): DRP1 + OPA1 vs. DRP1 and OPA1 = 0.9689 vs. 0.8667 and 0.9289]. Our study detected the first innovative combination of DRP1 and OPA1 to predict cognitive impairment in diabetes mellitus, which showed important clinical significance for better understanding the pathogenesis of cognitive impairment in diabetes mellitus.

Recent studies have shown that mitochondrial modification was regarded as a biological target of cell aging, and it mainly played a role by participating in energy production, oxidative stress, and regulating programmed cell death [36], which may be related to the injury of cardiopulmonary, nervous and musculoskeletal systems, thus producing adverse effects on the body. However, two studies in 2022 [37, 38] showed that exercise, nutrition and dietary supplements may affect the modification of mitochondria and thus delay the adverse effects on the body, which also provides a new idea for us to delay the cognitive impairment related to diabetes.

The results of this study suggested that DRP1 and OPA1 amplifications might be potential biomarkers for predicting cognitive impairment in diabetes mellitus. By detecting the amplification of DRP1 and OPA1 in patients, the mortality and poor functional outcome of cognitive impairment and peripheral neuropathy in diabetes mellitus may be intervened as early as possible. It is a promising therapeutic intervention for the secondary prevention of cognitive impairment in diabetes mellitus.

### Limitations

However, this study had some limitations. It was a single-center study with small sample size, leading to certain selection bias, which might also be the reason why the relationship between OPA1 and AD8 scores were not statistically significant. Second, since this was a cross-sectional study, the causal relationship and specific mechanisms between DRP1/OPA1 and cognitive impairment in diabetes were unclear. In addition, our study did not rule out other pathways such as oxidative stress and inflammatory responses for diabetes-related cognitive impairment.

## Conclusions

High DRP1 DNA expression and low OPA1 DNA expression have predictive value for cognitive deficits and may be promising biomarkers for MCI and dementia.

## Data Availability

The datasets used and/or analyzed during the current study are available from the corresponding author on reasonable request.
